# Longitudinal Capsulotomy in Hip Arthroscopy: A Safe and Feasible Procedure for Cam‐Type Femoracetabular Impingement

**DOI:** 10.1111/os.13041

**Published:** 2021-08-05

**Authors:** Qing‐Feng Yin, Long Wang, Tao Liang, Heng Zhao, Xue‐song Wang

**Affiliations:** ^1^ Department of Orthopedics The Second Hospital of Shandong University Jinan China; ^2^ Department of Orthopedics Chinese PLA General Hospital Beijing China; ^3^ Department of Orthopedics The First People's Hospital of Ningyang county Taian China; ^4^ Department of Sports Medicine Beijing Jishuitan Hospital Beijing China

**Keywords:** Femoracetabular impingement, Hip arthroscopy, Longitudinal capsulotomy

## Abstract

**Objective:**

To evaluate the surgical security, feasibility, and clinical efficacy of the longitudinal outside‐in capsulotomy in hip arthroscopic treatment for cam‐type femoracetabular impingement (FAI).

**Methods:**

We retrospectively reviewed patients with cam‐type FAI who underwent hip arthroscopy in our institute from January 2018 to June 2019. All hip arthroscopic procedures were performed by one experienced surgeon in the same manner, except the fashions of capsulotomy. Fifty six patients with mean age of 39.1 and mean body mass index (BMI) of 24.5 were categorized into two groups according to the fashions of capsulotomy. Twenty six cases with longitudinal outside‐in capsulotomy were categorized into Group L, and 30 cases with transversal interportal capsulotomy were categorized into Group T as the control group. The demographic parameters were retrieved from medical documents and compared between the two groups. Surgical outcome including overall surgical time, traction time, complications, visual analogue score (VAS), and intraoperative radiation exposure were compared to investigate the security and feasibility. Radiographic assessment, and functional outcome were compared between the two groups to determine the clinical efficacy of the longitudinal capsulotomy.

**Results:**

There was no significant difference in the demography and duration of follow‐up between the two groups. The overall surgical time demonstrated no significant difference between Group L and Group T (130.8 ± 16.6 min and 134.0 ± 14.7 min, *P* = 0.490). Significantly decreased traction time was found in Group L (43.2 ± 8.4 min and 62.2 ± 8.6 min, *P* < 0.001) compared to Group T. The Median of the fluoroscopic shot was 1 and 3 (*P* < 0.001). No major complications and reoperation were reported in both groups. The case of intraoperative iatrogenic injure was 0 (0%) and 6 (20%) in Group L and Group T respectively (*P* = 0.035), and the case of postoperative neurapraxia was 0 (0%) and 8 (26.6%) in Group L and Group T respectively (*P* = 0.017). The Median of postoperative VAS was 2 and 3 in Group L Group T (*P* = 0.002). The postoperative α angle was 42.3° ± 3.4° and 44.4° ± 3.5° in group L and group T respectively (*P* = 0.001). The postoperative iHOT‐12 score at final follow‐up was 79.3 ± 6.7 and 77.0 ± 7.9 respectively (*P* = 0.141).

**Conclusion:**

Longitudinal outside‐in capsulotomy with less radiation exposure, reduced traction time, and reduced complications could be a safe and feasible procedure in arthroscopic treatment for cam FAI. Its clinical efficacy was not worse compared with traditional interportal capsulotomy in short‐term follow‐up.

## Graphical abstract

The longitudinal outside‐in capsulotomy is a safe and feasible novel technique with less intraoperative fluoroscopy, reduced traction time, and decreased complications. Performing longitudinal capsulotomy could facilitate achieving sufficient visualization and complete correction of the cam lesion.

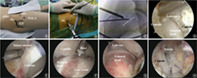

## Introduction

In recent years, hip arthroscopy has become the mainstream of surgical treatment for femoracetabular impingement (FAI)^1^. Being different from other joints, the hip joint enveloped by the thick and tenacious capsule, which provides the hip joint sufficient stability but also obstructed the procedure getting into the joint. Capsulotomy was the most important evolution in the process of hip arthroscopic techniques, and the most popular capsulotomy technique is the so‐called interportal capsulotomy which transversely connects the lateral portal and anterior portal on the capsule^2^. Capsulotomy could dramatically increase the visualization of arthroscopy and the mobility of instruments, which facilities the performing of complicated procedures such as labrum repair. However, the shortcomings of interportal capsulotomy should not be ignored, including intraoperative iatrogenic injury during portal establishing and traction‐related postoperative neurapraxia^3^. Traditional interportal capsulotomy was performed based on the portal establishment with Seldinger technology, and the iatrogenic injury and time consuming procedure are highly dependent on the surgeon's experience. Moreover, interportal capsulotomy usually transversally sections the iliofemoral ligament (IFL), which could result in potential iatrogenic hip instability and may have a negative effect on clinical outcomes ^4^, ^5^, ^6^.

With the recognition of the importance of capsule advancing, some surgeons practice the capsule preservation technique. Denist *et al*.^7^ proposed the peripheral compartment first technique characterized by decreased traction time, with starting hip arthroscopy from the peripheral compartment and then followed by the central compartmental procedure. Conaway *et al*.^8^ proposed the puncture capsulotomy technique. These techniques could decrease the damage to IFL and restore the integrity of the capsule, but much more skill is required compared with traditional interportal capsulotomy. More recently, Thaunat *et al*.^9^ proposed a novel technique of capsulotomy that starts from the peri‐capsular space and longitudinally split capsule between two branches of IFL in an outside‐in fashion without traction. Although the longitudinal fashion of capsulotomy could promise good visualization, sufficient space for practice, and ease of capsule closure, this procedure has not been popularly utilized, and the report on the clinical outcome of this procedure was limited. Comparing to traditional interportal capsulotomy, the superiority of longitudinal outside‐in capsulotomy in surgical security and clinical outcome following hip arthroscopy has not been well studied.

In our institute, interportal capsulotomy has been performed as a routine technique in hip arthroscopy for a long period. Recently, the longitudinal outside‐in capsulotomy technique was applied for cam‐type FAI. Therefore, in the current study, we reviewed patients who underwent hip arthroscopy diagnosed with cam‐type FAI. The purpose of this study was to: (i) introduce our practice of longitudinal capsulotomy in hip arthroscopy; (ii) investigate the surgical result and clinical outcome of hip arthroscopy with longitudinal capsulotomy; and (iii) compare longitudinal capsulotomy *vs* the traditional interportal capsulotomy in security, feasibility, and clinical efficacy.

## Patients and Methods

### Study Design and Participant

This study approved by the institutional review board (No. 2019LW016‐1) retrospectively reviewed consecutive patients who underwent hip arthroscopy between January 2018 and June 2019 in our database.

### Inclusion Criteria

Inclusion criteria were: (i) patients age between 18 and 60 years old; (ii) diagnosed with cam‐type FAI; (iii) underwent hip arthroscopy with capsulotomy in interportal or longitudinal fashion; and (iv) with outcome of minimum 1‐year follow‐up.

### Exclusion Criteria

Patients were excluded if they had: (i) Tönnis grade ≥ 2; (ii) hip dysplasia; (iii) presence of pincer deformity; (iv) inflammatory synovitis of the hip; (v) avascular necrosis of femoral head; and (vi) previous ipsilateral or contralateral hip surgery.

The medical records of 92 cases that met the inclusive criteria were screened and 25 cases were excluded for the presence of pincer deformity, two cases were excluded for hip dysplasia, four cases were excluded for inflammatory synovitis, two cases were excluded for avascular necrosis, and three cases were excluded for contralateral hip surgery. Finally, 56 cases with cam‐type FAI were enrolled in the present study with 25 males and 31 females. The average age of this cohort was 39.1 (range 18–59 years), and the mean body mass index (BMI) was 24.5 (range, 17.6–31.2). Twenty six cases with longitudinal outside‐in capsulotomy were categorized into Group L, and 30 cases with transversal interportal capsulotomy were classified into Group T as the control group.

### Indications for Surgery

The diagnosis of FAI was made by a senior surgeon according to the classical symptoms, physical examination, and radiologic information. Patients with symptom duration exceed 6 months, failure of conservative therapy, and positive finding of labral tear on MRI would be recommended to take hip arthroscopy.

### Surgical Procedure

#### Positon and Landmarks

**Fig. 1:**
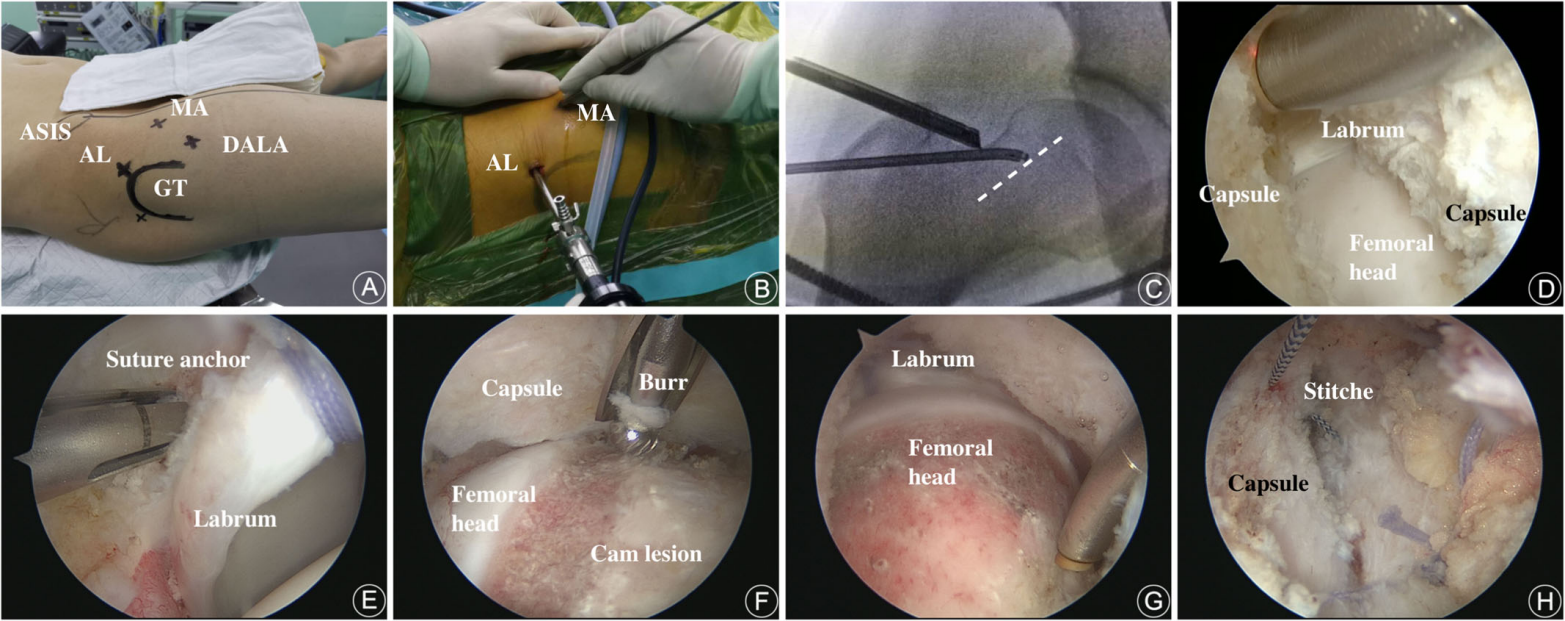
Surgical procedures of hip arthroscopy with longitudinal outside‐in capsulotomy for FAI. (A) Patient was prepared in a supine position on the fracture table as routine, and the landmarks were outlined. AL, anterolateral portal; ASIS, anterior superior iliac spine; DALA, distal anterolateral accessory portal; GT, great trochanter; and MA, mid‐anterior portal. (B) Viewing in the AL portal with a 30‐degree scope, instruments were introduced into the pre‐capsular space through the MA portal. (C) The location and direction of capsular incision (dash line) could be confirmed with fluoroscopy. (D) The capsular incision was extended to the labrum proximally and femoral neck distally. (E) The torn labrum was repair with suture anchors. (F) The peripheral compartment was comprehensively inspected and the cam lesion was identified and resected with a dynamic burr. (G) The cam lesion was completely addressed. (H) Capsular closure was performed with 2 simple stitches in a side‐to‐side fashion.

The standard setup of hip arthroscopy in the supine position with a fracture table and conventional instruments of arthroscopy were routinely utilized. The operative limb was placed in a neutral position of abduction‐adduction with 5–10 degrees of flexion, and the contralateral side was placed in 45 degrees of abduction position. The pudendal post was eccentrically positioned, and the feet were well‐padded and fixed in traction boots. The cutaneous outline of the anterior superior iliac spine (ASIS) and the great trochanter were marked before surgery, and then the anterolateral (AL) portal, mid‐anterior (MA) portal, and distal anterolateral accessory (DALA) portal were routinely marked. (Fig. 1A).

#### Portal Establishment and Capsulotomy

*Longitudinal Outside‐in Capsulotomy*. This procedure was performed without traction. A blunt trocar was introduced targeting the head–neck junction of the femoral head to establish the AL portal, and the fatty and fibrous tissue in front of hip capsule was identified with a 30‐degree scope. Instruments were introduced into the pre‐capsular space thorough the MA portal to triangulate with the same maneuver. The soft tissue in front of the capsule was cleaned, and the gluteal muscle, iliocapsularis muscle, and indirect head of rectus femoris were identified as the reference structure. A longitudinal capsular incision was made along the direction of IFL fiber paralleling to the axis of femora neck, and the fluoroscopy would be helpful in guiding for capsulotomy if necessary. The incision was extended to the labrum proximally and femoral neck distally (Fig. 1B–D).

*Transversal Interportal Capsulotomy*. Traction was applied and then the hip joint space exceeding 10 mm was confirmed with fluoroscopy. A 17G spinal needle was used to penetrate the joint capsule with the assistant of a C‐arm. and the AL portal was established using a cannulated dilator along a nitinol guidewire. And then, a 70‐degree scope was introduced as the viewing portal. The MA portal was established under visualization in the same method. Finally, an arthroscopic blade was used to make a capsular incision connecting AL and MA portal. Capsulotomy would be extended if necessary.

#### Exploration and Management in the Central Compartment

Scope and instruments were introduced into the central compartment of the hip joint with traction. The chondrolabral injure, ligamentum teres, and pathology on the acetabulum were identified and addressed. DALA portal was established for acetabular trimming and anchor implant. (Fig. 1E).

#### Exploration and Management in the Peripheral Compartment

The traction was released and the hip was flexed by 30–60 degrees. The peripheral compartment was comprehensively inspected and the cam lesion was identified, and then a 4.5 mm high‐speed arthroscopic burr (Smith & Nephew, Andover, MA) was used to make cam‐plasty. The intraoperative dynamic impingement test and fluoroscopy were used to identify the complete correction of cam lesion. (Fig. 1F–G).

#### Capsular Closure

**Fig. 2:**
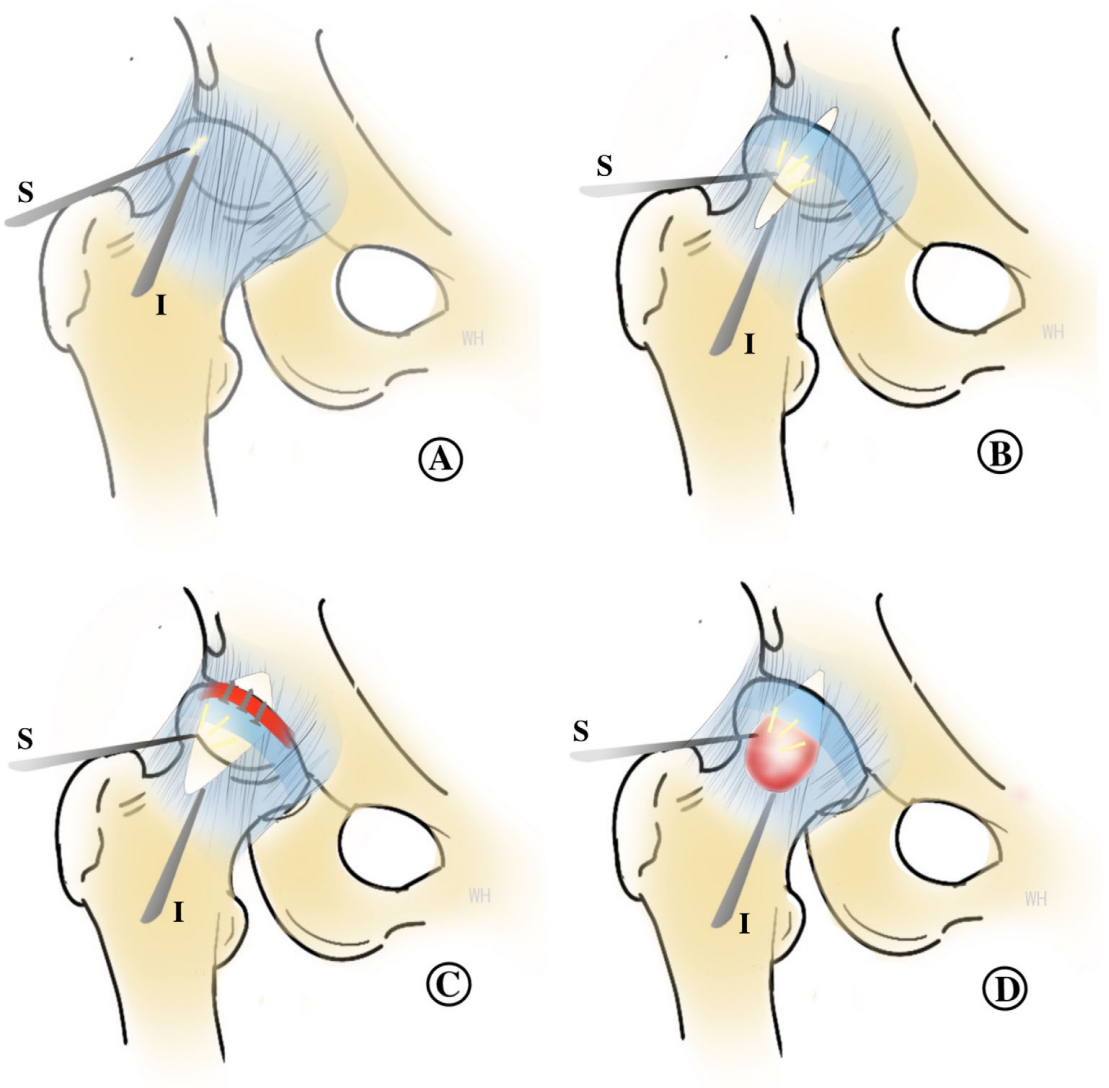
The diagram shows the main procedures of hip arthroscopy with longitudinal outside‐in capsulotomy (S, Scope; I, Instrument). (A) A 30‐degree scope viewing at AL portal, instruments were introduced into the pre‐capsular space through the MA portal. (B) A capsular incision was performed paralleling to the axis of the femoral neck and extended to the labrum proximally and femoral neck distally. (C) The limbs of proximal capsular incision were stretched to improve acetabular visualization and the torn labrum was refixed anchors. (D) The limbs of the distal capsular incision were stretched to improve visualization of the femoral head and the cam lesion was addressed using a dynamic burr.

At the end of procedure, two or three simple side to side stitches was made to close the capsule (Fig. 1H). The main procedures of hip arthroscopy with longitudinal capsulotomy was shown as the following schematic diagram. (Fig. 2).

### Postoperative Management

All patients followed the same protocol of postoperative analgesia. The oral nonsteroidal anti‐inflammatory drug was prescribed for 4 weeks for prophylaxis of heterotopic ossification. All patients followed the standard protocol of rehabilitation. Patients who received labral refixation or/and cam‐plasty were ambulated with crutches for 4 weeks.

### Data collection and Assessment of Outcomes

The surgical outcome including overall surgical time, traction time, intraoperative radiation exposure, complications, and postoperative pain were retrieved from the medical documents of patients. Radiographic parameters including α angle, lateral center edge angle, and Tönnis classification of osteoarthritis were assessed by two surgeons independently with Picture Archiving and Communication Systems (PACS), and the final result was made by a senior surgeon in cases of disagreement. Functional outcome was retrieved from the database of follow‐up. The definitive information of the measurement is described as follows.

#### Intraoperative Radiation Exposure

Intraoperative radiation exposure was defined as the number of fluoroscopic shots during surgery. The number of fluoroscopic shots during surgery was counted by a radiologist according to the images saved in the C‐arm X‐ray machine.

#### Complications

Intraoperative complications including iatrogenic cartilage or labral injury and breakage of instruments were review from the surgical video database. Postoperative complications including neurapraxias, infection, heterotopic ossification, deep venous thrombosis, and revision were documented in the medical record. Both intraoperative and postoperative complications were independently counted by a surgeon according to the documentary data.

#### Visual Analogue Score (VAS)

The visual analog scale is a widely used measurement for pain intensity. A continuous scale with a length of 10 cm, with the left end of the scale labeled 0 indicates no pain, and the right end labeled 10 indicates most severe pain. The location of marked between two ends represents the severity of pain ranged from 0 to 10. Postoperative pain was evaluated by an experienced nurse with the VAS score, and the highest score during postoperative 3 days was retrieved.

#### α Angle

The α angle was measured on the Dunn view radiography, which is the angle formed by the central axis of the femoral neck and the radius line where the femoral head loses its sphericity. The α angle was evaluated on the day before surgery and at 1‐month follow‐up. The cam lesion was defined with α ≥55°.

#### Lateral Center Edge Angle (LCEA)

The LCEA is formed by the vertical reference line to the line connecting the center of the femoral head and the most lateral edge of the acetabulum, which indicates the coverage of the acetabulum on the femoral head. The pincer lesion was defined with LCEA ≥38°.

#### International Hip Outcome Tool −12 (iHOT‐12) Score

The iHOT‐12 is the condensed version of the widely recognized International Hip Outcome Tool^10^. Each question was followed by a VAS range from 0 to 100, the patient was asked to answer each question by marking on the scale to reflect their limitation in this term. The iHOT‐12 score is calculated by averaging all scores. iHOT‐12 provides an overall assessment of the patient's hip function. The functional outcome was assessed with a patient‐reported outcome (iHOT‐12) on the day before surgery, 3 months, 6 months, and 12 months' follow‐up.

### Statistical Analysis

All data were analyzed using the SPSS 22.0 software (SPSS Inc., Chicago, IL, USA). Continuous variables were summarized with mean ± standard deviation or median and interquartile range (IQR). Continuous variables with normal distribution including age, BMI, α angle, LCEA, iHOT‐12, overall surgery time, and traction time were compared using a two‐sample t‐test. Quantitative data including the month of follow‐up, VAS, and intraoperative fluoroscopy which not follow normal distribution were compared by a two‐sample Wilcoxon rank‐sum test. Categorical variables including gender, Tönnis classification, and complications were presented with number and percentage and compared using the chi‐square test or the Fisher exact test. A *P*‐value of <0.05 was considered statistically significant.

## Results

### General Results

Demographic data showed no significant difference between the two groups. The male/female ratios were 12/14 and 13/17 in Group L and Group T respectively (*P* = 0.832). The average age was 38.2 ± 11.1 and 40.1 ± 9.9 in Group L and Group T respectively (*P* = 0.530). The mean BMI was 24.7 ± 3.5 and 24.1 ± 3.3 in Group L and Group T respectively (*P* = 0.448). The Median of follow‐up duration was 16 months and 18 months in group L and group T respectively (*P* = 0.107). Labral refixation was performed for each patient with labral tear. No major complications and reoperation were reported in both groups (Table 1).

**TABLE 1:** Demographic data

Characteristics	Group L (N = 26)	Group T (N = 30)	*P*‐value
Gender (M/F)	12/14	13/17	0.832
Age (year)	38.2 ± 11.1	40.1 ± 9.9	0.530
Body mass index (kg/m^2^)	24.7 ± 3.5	24.1 ± 3.3	0.448
Tönnis classification (grade 0/1)	21/5	23/7	0.709
Lateral center edge angle (°)	31.5 ± 3.4	30.8 ± 3.1	0.386
Preoperative α angle (°)	60.8 ± 4.2	61.2 ± 4.7	0.670
Preoperative iHOT‐12 score	38.9 ± 13.7	41.1 ± 15.6	0.514
Follow‐up (month), median (IQR)	16 (14, 18)	18 (15, 22)	0.107

Data are expressed as mean ± SD, median (range), and number (ratios).

iHOT‐12, International Hip Outcome Tool‐12; IQR, interquartile range.

### Surgical Results

#### Overall Surgery Time

No significant differences in the overall surgery time were found between Group L and Group T (130.8 ± 16.7 min and 134.0 ± 14.7 min, *P* = 0.490).

#### Traction Time

The mean traction time of hip arthroscopy in Group L and Group T was 43.2 ± 8.4 min and 62.2 ± 8.6 min, respectively. Performing longitudinal outside‐in capsulotomy significantly shortened the traction time by 19 min (30.54%) during hip arthroscopy (*P* < 0.001).

#### Intraoperative Fluoroscopy

It is apparent that the radiation exposure with longitudinal outside‐in capsulotomy was much lower than that with transversal interportal capsulotomy, Median of intraoperative fluoroscopy shots was 1 and 3, respectively. Performing longitudinal outside‐in capsulotomy significantly reduced the radiation exposure by 66.7% compared to transversal interportal capsulotomy (*P* < 0.001).

### Radiological Results

#### Tönnis Classification

Two groups have no significant difference in the respect of osteoarthritis (*P* = 0.709), and there are five cases in Group L and seven cases in Group T present mild degeneration on radiography (Tönnis grade 1) respectively.

#### α Angle

The postoperative α angle was significantly smaller than that with transversal capsulotomy (42.3° ± 3.4° and 44.4° ± 3.5°, *P* = 0.001). Patients who underwent hip arthroscopy with longitudinal capsulotomy get a more complete cam correction and reduced postoperative α angle by 5% compared to that with transversal capsulotomy. The measurement of preoperative α angle was 60.8 ± 4.2 and 61.2 ± 4.7 in Group L and Group T respectively (*P* = 0.670). The change of α angle was also compared and that was 18.3 ± 4.7 and 16.9 ± 4.3 in Group L and Group T respectively (*P* = 0. 221).

#### Lateral Center Edge Angle (LCEA)

**Fig. 3:**
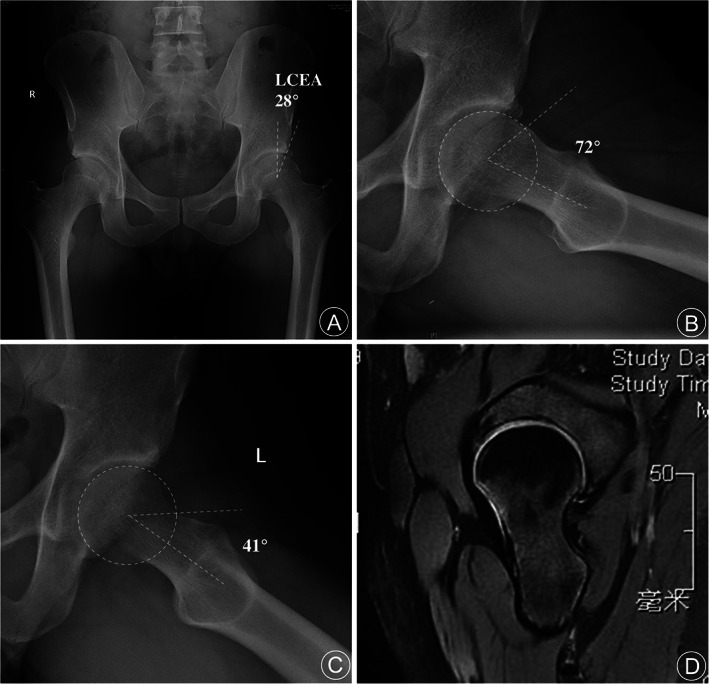
The radiologic outcome of a 32‐year‐old male patient with cam FAI underwent hip arthroscopy with longitudinal outside‐in capsulotomy. (A) The preoperative anteroposterior pelvic view with normal acetabular coverage (LCEA 28°) and os acetabuli on the left hip. (B) The preoperative Dunn view of the left hip with a significant bump of cam lesion in head–neck junction (α angle 72°). (C) The postoperative Dunn view of the left hip with the normal off‐set of head–neck junction established (α angle 41°). (D) The hip MRI image at 1‐year follow‐up shows that the capsule completely healed, and there is no adhesion and effusion.

The measurement of preoperative LCEA was 31.5 ± 3.4 and 30.8 ± 3.1 in Group L and Group T respectively (*P* = 0.386) (Fig. 3A–C).

### Functional Results

#### Postoperative Pain

The Median of VAS in Group L and Group T was 2 and 3. Performing longitudinal capsulotomy significantly reduced the postoperative pain by 33.3% compared to the transversal interportal capsulotomy (*P* = 0.002) (Table 2).

**TABLE 2:** Surgical, radiographic, and functional outcomes

Characteristics	Group L (N = 26)	Group T (N = 30)	*P*‐value
Overall surgical time (min)	130.8 ± 16.6	134.0 ± 14.7	0.490
Traction time (min)	43.2 ± 8.4	62.2 ± 8.6	<0.001
Intraoperative fluoroscopy, median (IQR)	1 (1, 2)	3 (2, 3)	<0.001
Intraoperative complication	0 (0%)	6 (20%)	0.035
Postoperative complication	0 (0%)	8 (26.6%)	0.017
VAS for pain, median(IQR)	2 (1, 3)	3 (2, 4)	0.002
Postoperative iHOT‐12 score	79.3 ± 6.7	77.0 ± 7.9	0.141
Improvement of iHOT‐12 score	40.3 ± 11.6	35.9 ± 15.5	0.228
Postoperative α angle (°)	42.3 ± 3.4	44.4 ± 3.5	0.001
Change of α angle (°)	18.3 ± 4.7	16.9 ± 4.3	0.221

Values are expressed as mean ± SD, median (range), and number(percentage).

iHOT‐12, International Hip Outcome Tool‐12; IQR, interquartile range; VAS, Visual Analogue Score.

#### iHOT‐12 Score

The preoperative iHOT‐12 score shows no significant difference between the two groups, and that was 38.9 ± 13.7 and 41.1 ± 15.6 in Group L and Group T respectively (*P* = 0.514). Hip arthroscopy dramatically improved iHOT‐12 of patients in both groups, and the postoperative iHOT‐12 score at final follow‐up was 79.3 ± 6.7 and 77.0 ± 7.9 in Group L and Group T respectively (*P* = 0.141).

### Complications

There are no iatrogenic chondrolabral injure (0%) and no postoperative neurapraxia (0%) was reported in Group L. In contrast to that, there are six cases (20.0%) of iatrogenic chondrolabral injure and eight cases (26.6%) of transient neurapraxia (fully recovered in 2 weeks) were recorded in Group T. The incidence of intraoperative iatrogenic chondrolabral injure and postoperative neurapraxia was significantly reduced by performing performing longitudinal capsulotomy. (*P* = 0.035 and 0.017, respectively.)

## Discussion

The current study found that performing the longitudinal capsulotomy in an outside‐in fashion did not consume additional time to accomplish hip arthroscopy but significantly reduced the traction time and radiation exposure compared with the conventional technique. As a result, the case of complication and postoperative pain was significantly decreased. Additionally, performing longitudinal capsulotomy could facilitate the complete correction of the cam lesion. However, the influence of longitudinal capsulotomy on patient‐reported outcomes in short‐term follow‐up was not obvious.

### Traction Time and Traction‐Related Complications

The most important finding of the present study is that performing longitudinal outside‐in capsulotomy could reduce traction time and traction‐related complications of hip arthroscopy. The reported complication rate of hip arthroscopy varies from 0.5% to 8%, and most of them are traction related^3^, ^11^, ^12^, ^13^. Frandsen *et al*.^14^ reported that up to 74% of patients complained of some kind of traction‐related problems after hip arthroscopy, and neurapraxia is the most commonly reported. Kern *et al*. ^15^ reported the incidence of nerve injury after hip arthroscopy could be up to 13% and the traction related neurapraxia was underestimated previously. Bailey *et al*.^16^ reported the mean traction time was 46.5 min, and a longer traction time and a greater traction force could result in groin numbness and pudendal neurapraxia. In the present study, all eight cases of transient neurapraxia reported came from Group with transversal capsulotomy. A possible explanation for this result was that longitudinal capsulotomy could be performed without traction, thus the traction time was dramatically reduced by around 20 min and only 43.2 min on average traction lasted in the present study. Moreover, Röling *et al*.^17^ found the traction force could significantly drop after breakage of the vacuum seal labrum and additional capsulotomy. Another possible reason we speculate was that the traction force could be dramatically decreased after capsulotomy. Additionally, to prevent the traction‐related complication we strictly followed the recommendations include minimizing the traction force, limiting the traction time, and using a well‐padded perineal post^18^, ^19^. Another finding of the present study endorses the traction advantage of longitudinal capsulotomy is that patients who underwent hip arthroscopy with longitudinal capsulotomy felt more comfortable and reported milder pain than those with traditional capsulotomy. In accordance with this interesting finding, Martin *et al*.^20^ demonstrated that tissue damage could be decreased with less traction.

### Radiation Exposure

Another finding of this study is that radiation exposure in hip arthroscopy could be decreased by performing longitudinal capsulotomy. The utilization of fluoroscopy is a near essential procedure for traditional hip arthroscopy, which could help surgeons in performing portal establishment and lesion correction. Meanwhile, the impairment of radiation for the patient and the surgical team could not be ignored. Seijas^21^ and Gaymer^22^ independently reported the mean exposure time was around 20 s in each hip arthroscopic procedure. Budd *et al*.^23^ reported that the mean radiation time was 66 s in their study. The intraoperative radiation exposure could be related to the surgical fashion and experience of the surgeon. The exposure time in the present study is much shorter than that previously reported, and only several fluoroscopy shots were taken in each hip arthroscopic procedure. This inconsistency may be due to that performing longitudinal capsulotomy in an outside‐in fashion with direct visualization further reduced the assistance of fluoroscopy. Moreover, all procedures were performed by an experienced surgeon, and fluoroscopy was used at critical steps in the present study.

### Feasibility of Practice

Even with less assistance of fluoroscopy performing longitudinal outside‐in capsulotomy reduced the incidence of iatrogenic chondrolabral injure and improved the resection of the cam lesion. Traditionally, the labrum and cartilage could not be seen during portal establishment, and penetration of labrum and cartilage scuffing was common in interportal capsulotomy. In contrast, performing longitudinal capsulotomy in an outside‐in fashion could provide direct visualization for all procedures, and the iatrogenic chondrolabral injury could be almost eliminated. Additionally, longitudinal capsulotomy could provide ideal visualization for the exposure of the cam lesion, especially the distally located one. It could facilitate the complete resection of the cam lesion and improve the postoperative outcome and reduce the need reoperation of hip arthroscopy.

### Capsule Preservation and Outcomes

The function of the iliofemoral ligament and the clinical benefit of restoring intact capsule was underlined, meanwhile, the shortcoming of interportal capsulotomy has been noted. Fagotti et.al^24^ found more than half of the width of the IFL could be damaged after interportal capsulotomy. Several studies indicated conventional interportal and T‐shaped capsulotomy could significantly decrease the strength of the iliofemoral ligament and affect the stability of the hip joint^25^, ^26^, ^27^. Bolia^28^ found superior outcomes could be expected in patients with capsular closure compared with unrepaired capsulotomy. Capsular closure was suggested to be performed for large interportal capsulotomies or T‐capsulotomy^29^, ^30^, ^31^. But there are few studies that reported whether capsular closure should be performed after longitudinal outside‐in capsulotomy, except one study, Thaunat found capsular closure after longitudinal outside‐in capsulotomy might positively affect the final outcome^32^. It is rational that patient that had longitudinal capsulotomy with the function of the iliofemoral ligament and the integrity of the capsule maximally retained would expect to get a better outcome. In our practice, most patients with longitudinal capsulotomy could achieve well‐healing of the capsule in the short‐term follow‐up with MRI (Fig. 3D). However, in the present study, the divergence in patient‐reported outcomes between the two groups was not statistically significant. One explanation for this result could be that all procedures were performed by one experienced surgeon, and the capsule closure was performed in both groups. Another reason may be that the duration of follow‐up is not long enough to distinguish the superiority of longitudinal capsulotomy.

Limitations of the present study include the retrospective nature of the analysis. Patients with pincer deformity were excluded because of the high transition rate to T capsulotomy, which may limit the generalization of the findings. Even though a positive result was acheived, limitations that should not be ignored are the sample size of the present study, which is relatively small and the duration of follow‐up is relatively short. Although Wolfson^33^ and Nwachukwu^34^ found most patients could achieve minimal clinically important difference or a substantial clinical benefit at postoperative 6 months. We insist that further study with a long duration of follow‐up was needed to identify the clinical efficiency of longitudinal capsulotomy.

Besides the limitations mentioned above, several features of the present study should be noted. First, the difficulty or inconvenience encountered when making rim resection and anchor implant with longitudinal capsulotomy should not be ignored, and we prefer to add a DALA portal to address this problem. Second, the clinical result came from one experienced surgeon who has practiced hundreds of hip arthroscopies with traditional capsulotomy. We do not consider that a surgeon with less experience could easily reproduce this result. Third, specific complications and underneath risks related to longitudinal capsulotomy could be encountered in the future. We have recorded one case with the indirect head of the femoral rectus injured intraoperatively. Fortunately, the injury was noted and completely repaired with suture, and the patient has no postoperative complications. Additionally, the impairment of longitudinal capsulotomy to the orbicular zona has not been investigated.

### Conclusion

Longitudinal outside‐in capsulotomy with less radiation exposure, reduced traction time, and deceased complications could be a safe and feasible procedure in arthroscopic treatment for cam FAI. Its clinical efficacy is not worse compared with traditional interportal capsulotomy in short‐term follow‐up.
